# Comparison of Mitochondrial Superoxide Detection Ex Vivo/In Vivo by mitoSOX HPLC Method with Classical Assays in Three Different Animal Models of Oxidative Stress

**DOI:** 10.3390/antiox8110514

**Published:** 2019-10-28

**Authors:** Sanela Kalinovic, Matthias Oelze, Swenja Kröller-Schön, Sebastian Steven, Ksenija Vujacic-Mirski, Miroslava Kvandová, Isabella Schmal, Ahmad Al Zuabi, Thomas Münzel, Andreas Daiber

**Affiliations:** 1 Center for Cardiology, Department of Cardiology, Molecular Cardiology, University Medical Center, 55131 Mainz, Germany; sanelakalinovic@gmail.com (S.K.); matzeoelze@aol.com (M.O.); swenja.kroeller-schoen@gmx.de (S.K.-S.); sesteven@uni-mainz.de (S.S.); ksenija.vujacic.mirski@gmail.com (K.V.-M.); miroslava.kvandova@gmail.com (M.K.); isabella.schmal@gmx.de (I.S.); aalzuabi@students.uni-mainz.de (A.A.Z.); tmuenzel@uni-mainz.de (T.M.); 2 Partner Site Rhine-Main, German Center for Cardiovascular Research (DZHK), Langenbeckstr. 1, 55131 Mainz, Germany

**Keywords:** oxidative stress, mitochondrial superoxide detection, mitoSOX, hypertension, diabetes, nitrate tolerance

## Abstract

Background: Reactive oxygen and nitrogen species (RONS such as H_2_O_2_, nitric oxide) are generated within the organism. Whereas physiological formation rates confer redox regulation of essential cellular functions and provide the basis for adaptive stress responses, their excessive formation contributes to impaired cellular function or even cell death, organ dysfunction and severe disease phenotypes of the entire organism. Therefore, quantification of RONS formation and knowledge of their tissue/cell/compartment-specific distribution is of great biological and clinical importance. Methods: Here, we used a high-performance/pressure liquid chromatography (HPLC) assay to quantify the superoxide-specific oxidation product of the mitochondria-targeted fluorescence dye triphenylphosphonium-linked hydroethidium (mitoSOX) in biochemical systems and three animal models with established oxidative stress. Type 1 diabetes (single injection of streptozotocin), hypertension (infusion of angiotensin-II for 7 days) and nitrate tolerance (infusion of nitroglycerin for 4 days) was induced in male Wistar rats. Results: The usefulness of mitoSOX/HPLC for quantification of mitochondrial superoxide was confirmed by xanthine oxidase activity as well as isolated stimulated rat heart mitochondria in the presence or absence of superoxide scavengers. Vascular function was assessed by isometric tension methodology and was impaired in the rat models of oxidative stress. Vascular dysfunction correlated with increased mitoSOX oxidation but also classical RONS detection assays as well as typical markers of oxidative stress. Conclusion: mitoSOX/HPLC represents a valid method for detection of mitochondrial superoxide formation in tissues of different animal disease models and correlates well with functional parameters and other markers of oxidative stress.

## 1. Introduction

Oxidative stress was identified as a hallmark of almost all cardiovascular and neurodegenerative diseases [1,2,3]. The term oxidative stress describes a condition that is either characterized by increased generation of reactive oxygen and nitrogen species (RONS) and/or dysregulated cellular antioxidant defense mechanisms (e.g., decreased expression of central antioxidant enzymes). This will ultimately cause that tissue, plasma or intercellular fluid run out of low molecular weight antioxidant compounds leading to detrimental alterations of cellular redox state [4,5]. The most abundant biological RONS include superoxide radical, hydrogen peroxide, hydroxyl radical, carbon-centered peroxides and peroxyl radicals, nitric oxide radical (NO∙), nitrogen dioxide radial, peroxynitrite, and hypochlorite. Some of these species were reported to act as cellular messengers and participate in redox signaling mechanisms [6,7,8,9]. Nitric oxide (NO∙) for instance acts as an important vasodilator and inhibitor of platelet activation via formation of nitrosyl-iron enzyme complexes [10] but also provides the basis for wide-spread signaling mechanisms by S-nitros(yl)ation of protein thiol groups as exemplified by suppression of apoptosis by S-nitros(yl)ation of caspases [11]. The superoxide radical anion (O_2_∙^−^) can be formed from different sources such as xanthine oxidase, NADPH (nicotinamide adenine dinucleotide phosphate) oxidases, uncoupled NO synthases and the mitochondrial respiratory chain, and represent in many ways a direct antagonist of nitric oxide as shown by the famous experiment by Gryglewski et al. [12]. The existence of superoxide dismutases (mitochondrial MnSOD and cytosolic/extracellular Cu, Zn-SOD) implies that superoxide may be harmful for the cell [13], which is supported by the fact that deficiency in MnSOD is lethal [14,15] and deficiency in Cu, Zn-SOD renders mice susceptible to cardiovascular and neuronal complications [16,17,18].

Since the transition between oxidative stress and redox signaling is a thin line [19,20,21,22], the exact knowledge of the identity of formed species, the cellular and subcellular localization of their formation, as well as their time duration and concentration, are of high clinical and pharmacological importance. With our previous work, we have stressed that the traditional or old RONS assays are not necessarily inferior as compared to up-to-date or even cutting-edge assays and at least have their specific features and application spectra [23]. With the present studies, we evaluated the specific probe for mitochondrial superoxide formation, mitochondria-targeted, triphenylphosphonium-linked hydroethidium (mitoSOX), for its usefulness in the detection of mitochondrial superoxide formation ex vivo/in vivo and compared this high-performance/pressure liquid chromatography (HPLC)-based assay with two alternative/traditional methods. For this purpose, we used three different animal models with well established oxidative stress burden, namely diabetic [24,25], hypertensive [26,27] and nitrate tolerant rats [28,29]. Given the recent report by Xiao & Meierhofer raising doubts about the reliable use of hydroethidium-based probes for superoxide detection in cell culture [30], we think that our present data are of interest for all researchers using hydroethidium-based probes, but especially mitoSOX, in cell culture, isolated mitochondria and tissue samples.

## 2. Materials and Methods 

### 2.1. Chemicals

**Figure 1:**
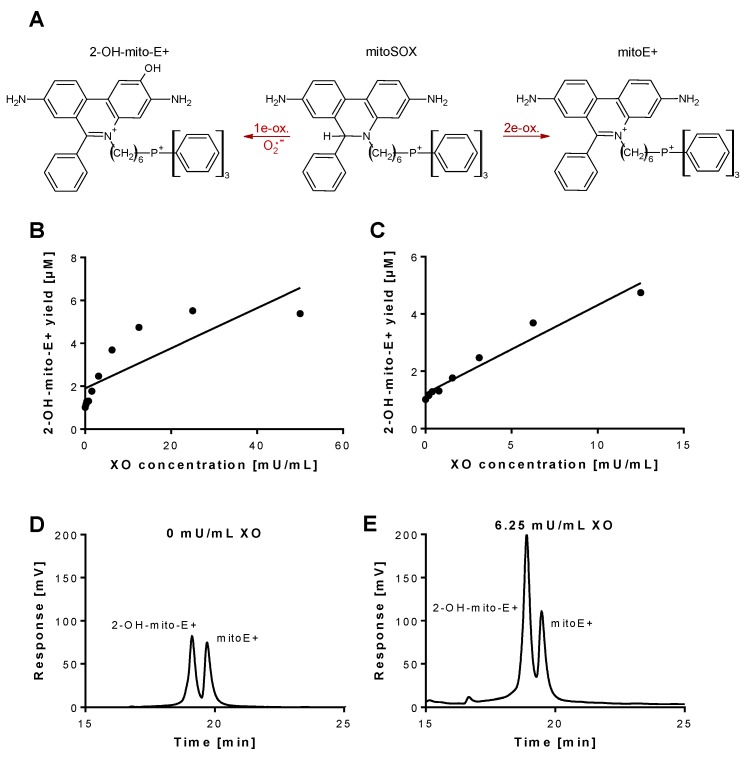
Detection of superoxide generation by xanthine oxidase by mitoSOX HPLC. (**A**) Structures of mitoSOX and its oxidation products 2-OH-mito-E+ (left) and mitoE+ (right). (**B**,**C**) The yield of the superoxide-specific mitoSOX oxidation product 2-OH-mito-E+ in dependence of the XO concentration (0–50 mU/mL). The reaction solution contained 0.1 M potassium phosphate buffer at pH 7.4 and 1 mM hypoxanthine and was incubated for 30 min at 37 °C. (**D**,**E**) Representative chromatograms are shown for the control without XO and the 6.25 mU/mL XO concentration. A single data point was obtained for each XO concentration. HPLC: high-performance/pressure liquid chromatography; mitoSOX: mitochondria-targeted fluorescence dye triphenylphosphonium-linked hydroethidium.

MitoSOX was purchased from Invitrogen/Thermo Fischer Scientific (Waltham, MA, USA). According to the vendors information, mitoSOX dye in pure form is stable about six months at room temperature. MitoSOX oxidation product standards, triphenylphosphonium ethidium (mitoE+) and triphenylphosphonium 2-hydroxyethidium (2-OH-mito-E+) were synthesized according to published protocols (for structures see Figure 1A) [31]. Briefly, 2-OH-mito-E+ was prepared by reacting mitoSox with Fremy’s salt using two moles of Fremy’s salt (considering dissociation in water into two molecules of nitrosodisulfonate, NDS) per one mole of mitoSOX. Accordingly, 50 µL of l mM solution of Fremy’s salt in 0.5 M phosphate buffer at pH 7.4 was mixed with 10 µL of 5 mM mitoSOX in dimethyl sulfoxide (DMSO) and 1 mL of 0.5 M phosphate buffer with 5% acetonitrile and incubated for 1 h at room temperature. MitoE+ was prepared by reacting mitoSOX with chloranil: 25 µL of 2 mM solution of chloranil in methanol was mixed with 10 µL of 5 mM mitoSOX in DMSO and 1 mL of 0.5 M phosphate buffer with 5% acetonitrile and incubated for 4 h at room temperature.

### 2.2. Animals and Treatment

The rats were investigated in accordance with the Guide for the Care and Use of Laboratory Animals as adopted by the U.S. National Institutes of Health and all animal experiments were approved by the Ethics Committee of the University Hospital Mainz and the Landesuntersuchungsamt Rheinland-Pfalz (Koblenz, Germany; permit number: 23 177-07/G 18-1-001). For surgery, we anesthetized the rats with isoflurane (implantation of osmotic pumps for angiotensin-II (AT-II) or nitroglycerin (GTN) infusion) or ketamine/xylazine (streptozotocin (STZ) injection), in order to minimize the suffering of the animals. Rats were killed under isoflurane anesthesia by exsanguination. In total, 16 male Wistar Rats were used (age: 6 weeks; weight: 300 g; purchased from Charles River Laboratories, Sulzfeld, Germany). Diabetes mellitus type 1 in rats was induced by injection of a single STZ dose into the vena dorsalis penis (dose: 60 mg/kg s.c.; solvent: 5 mM pH 4.5 citrate buffer) [24,32]. Sham treatment of control animals consisted of solvent injection. Diagnosis of diabetes was performed by determination of whole blood glucose levels with a dilution of blood from STZ-treated rats by 1:5 in NaCl solution by applying a small blood volume to the ACCU-CHEK Sensor system (Roche Diagnostics GmbH, Mannheim, Germany). We have previously shown that STZ treatment represents a reliable type 1 diabetes model since insulin co-therapy completely reversed and prevented the typical biological and biochemical complications observed in hyperglycemia (e.g., vascular dysfunction and oxidative stress) [33]. For induction of hypertension, rats were treated with either AT-II (1.0 mg/kg/d) or solvent (0.9% NaCl) for 7 d, as described previously [26,34]. For induction of nitrate tolerance, rats were equipped with micro-osmotic pumps (model 2001 from Alzet, Cupertino, CA, USA) containing an ethanolic solution of 450 mM GTN or pure ethanol as the solvent control with an average infusion rate of 6.6 µg/kg/min for 4 d, as described previously [35,36]. After 8 weeks (STZ), 7d (AT-II) or 4d (GTN) of total treatment duration, animals were killed under isoflurane anesthesia by transection of the diaphragm and removal of the heart and thoracic aorta for isometric tension studies, dot blot analysis, chemiluminescence and mitoSOX-based analyses. For protein expression also the abdominal part and the arch of the aorta were used.

### 2.3. Isometric Tension Recording Experiments

The vasodilation induced by acetylcholine (ACh, endothelium-dependent) and nitroglycerin (GTN, endothelium-independent) as well as the response to the vasoconstrictors KCl and phenylephrine were determined with isolated rat aortic ring segments with intact endothelium, as described previously [24,36]. The aortic ring segments were fixed in organ chambers for measurement of isometric tension and were then subjected to bolus concentrations of KCl (up to 80 mM) in order to test the vascular integrity of the smooth muscle and to “train” their vasoconstrictory function. Next, the aortic ring segments were pre-constricted with the α-agonist phenylephrine (PheE, 1 µM) to 50–60% of the tone induced by KCl and after reaching a stable tonus plateau, the vasodilator ACh was added by half-logarithmic cumulative concentration steps. After adding the maximal concentration of ACh (3.3 µM), two wash-out steps were performed, pre-constriction by PheE was induced and after reaching a stable tonus plateau, the vasodilator GTN was added by half-logarithmic cumulative concentration steps up to the highest concentration of 33 µM. 

### 2.4. Detection of Oxidative Burst in Whole Blood and Mitochondrial Reactive Oxygen Species (ROS) Formation in Aorta 

Leukocyte-dependent oxidative burst is a read-out for leukocyte-dependent hydrogen peroxide formation (mainly by phagocyte-type NADPH oxidase, Nox2). Hydrogen peroxide is converted by myeloperoxidase to highly reactive oxygen-metal complexes that lead to oxidation of L-012 (8-amino-5-chloro-7-phenylpyrido[3,4-d]pyridazine-1,4-(2H,3H)dione sodium salt) to an intermediate that by chemical reaction emits energy in the form of chemiluminescent light. Oxidative burst was measured in fresh citrate blood upon dilution 1:50 and stimulation with zymosan A (50 µg/mL) as well as phorbol ester dibutyrate (10 µM) in PBS buffer containing Ca^2+^/Mg^2+^ (1 mM) by L-012 (100 µM) enhanced chemiluminescence (ECL) using a Mithras^2^ chemiluminescence plate reader (Berthold Technologies, Bad Wildbad, Germany) [25,37]. L-012 was purchased from Wako Pure Chemical Industries (Osaka, Japan). Vascular mitochondrial ROS formation was determined using mitoSOX (1 µM)-dependent fluorescence microtopography in aortic cryo-sections after thawing and removing the tissue Tek resin as described [38]. A Zeiss Axiovert 40 CFL microscope with Zeiss lenses and equipped with an Axiocam MRm camera (Zeiss Microscopy, Jena, Germany) were used for the detection of red fluorescence, which originates from cellular ROS formation and subsequent mitoSOX oxidation in the tissue sections. The intensity of this fluorescence signal, which is based on oxidized mitoSOX products, was quantified by densitometry. Green staining originates from basal laminae autofluorescence.

### 2.5. Dot Blot Analysis

Analysis of total protein homogenates and of plasma samples was performed by dot blot as previously described [39,40]. Briefly, 100 µL (0.5 µg/µL protein based on Bradford analysis) of the heart homogenate or EDTA (ethylenediaminetetraacetic acid) plasma were bound to a nitrocellulose membrane (Protran BA85 (0.45 µm), Schleicher & Schuell, Dassel, Germany) using a vacuum Dot-Blot system (Minifold I, Schleicher & Schuell, Dassel, Germany). We washed each slot twice with 200 µL PBS (phosphate-buffered saline) before and after protein transfer. The membrane was dried for 60 min at 60 °C. Equal loading of protein amounts per dot was then verified by staining the membrane with Ponceau S (Sigma-Aldrich, St. Louis, MO, USA). Next, the membrane was incubated with blocking buffer and then primary antibody in blocking buffer according to the supplier’s instructions. Protein tyrosine nitration was detected using a specific antibody for 3-nitrotyrosine (mouse monoclonal, 3-NT, 1:1000, Upstate Biotechnology, Lake Placid, NY, USA) and lipid peroxidation using specific antibodies for malondialdehyde (MDA)-positive proteins (1:1000, Calbiochem, Darmstadt, Germany) or 4-hydroxynonenal (4-HNE)-positive proteins (mouse monoclonal, 1:1000, Percipio Biosciences, Foster City, CA, USA) in heart homogenate or EDTA plasma. Inflammation was detected using a specific antibody for interleukin-6 (anti IL-6 rabbit antibody, abcam, ab6672, Cambridge, UK) in heart homogenate or EDTA plasma. Positive bands were detected by enhanced chemiluminescence after incubation with a peroxidase-coupled secondary antibody (GAM-POX and GAR-POX, 1:10,000) (Vector Laboratories, Burlingame, CA, USA). We performed the steps for incubation and washing as described in the manual provided by the manufacturer. The quantitative evaluation of the staining signal of each dot was conducted using the Super Signal ECL kit from Thermo Scientific (Waltham, MA, USA) using a ChemiLux Imager (CsX-1400M, Intas, Göttingen, Germany) and Gel-Pro Analyzer software version 3 (Media Cybernetics, Bethesda, MD, USA).

### 2.6. Detection of Mitochondrial Superoxide Formation by mitoSOX HPLC Method and Plate Reader Assay in Isolated Heart Mitochondria

Mitochondrial oxidative stress and superoxide was also measured by a modified HPLC-based method to quantify triphenylphosphonium-linked 2-hydroxyethidium (2-OH-mito-E+) levels as previously described [31]. Briefly, cardiac tissues underwent homogenization in HEPES buffer (composition in mM: 50 HEPES, 70 sucrose, 220 mannitol, 1 EGTA (ethylene glycol-bis(β-aminoethyl ether)-N,N,N′,N′-tetraacetic acid), and 0.033 bovine serum albumin) and centrifugation at 1500× *g* for 10 min at 4 °C, followed by another centrifugation step of the supernatant at 2000× *g* for 5 min (pellets were not used). Next, centrifugation of the supernatant at 20,000× *g* for 20 min was applied, the pellet was collected and a suspension in 1 mL of HEPES buffer was prepared. The suspension was centrifuged again at 20,000× *g* for 20 min, but this time a suspension of the pellet was prepared in 1 mL of Tris buffer (composition in mM: 10 Tris, 340 sucrose, 100 KCl, and 1 EDTA). The resulting mitochondria-enriched suspensions containing 5–10 mg/mL of total protein (according to Lowry assay) were kept at 0 °C, were all adjusted to a similar protein content (based on the lowest determined concentration) and were further diluted in 0.5 mL of PBS buffer containing mitoSOX (5 µM) (final protein concentration: 0.1 mg/mL) and then incubated for 15 min at 37 °C. After the incubation step 50% *v/v* of acetonitrile was added in order to destroy the mitochondrial membrane and extract the mitoSOX oxidation products, samples were subjected to centrifugation and the resulting supernatant was subjected to HPLC analysis (100 µL per sample injection). The HPLC system was purchased from Jasco (Groß-Umstadt, Germany) with a typical composition (control unit, two pumps for high pressure gradient, high pressure mixer, UV/V is and fluorescence detectors, and an autosampler (AS-2057 plus with 4 °C cooling device). Generation of gas bubbles from the solvents that can cause an unstable detection baseline were prevented using a degasser unit. For separation of the product and reactant mixtures, a reversed-phase column was used (C_18_-Nucleosil 100-3 (125 × 4 mm), Macherey & Nagel, Düren, Germany). Optimal separation was achieved by application of a high pressure gradient with acetonitrile as the organic/nonpolar component and citrate buffer as the aqueous/polar component (50 mM, pH 2.2) of the mobile phase. The following percentages of the organic solvent were applied: 0 min, 22%; 10 min, 50%; 22 min, 63%; 23–25 min, 100%; 25–27 min, 22%. The flow was 0.5 mL/min and mitoSOX was detected by its absorption at 360 nm whereas mitoE+ and 2-OH-mito-E+ were detected by fluorescence (Ex. 500 nm/Em. 580 nm). The HPLC mitoSOX assay was also used for testing the linearity of 2-OH-mito-E+ product formation over a wide range of xanthine oxidase concentrations (0–50 mU/mL) and the effects of inhibitors oxypurinol (100–600 µM), Cu, Zn-SOD (400–1000 U/mL) and PEG-SOD (superoxide dismutase-polyethylene glycol) (200–600 U/mL). The reaction solution contained 0.1 M potassium phosphate buffer at pH 7.4 and 1 mM hypoxanthine and was incubated for 30 min at 37 °C. 

The mitochondrial supernatant was also used for the plate reader assay. Here, 200 µL of supernatant were pipeted in the 96 well black plate (Berthold Technologies, Bad Wildbad, Germany), and the fluorescence was measured by Mithras^2^ chemiluminescence/fluorescence plate reader with double monochromator (Berthold Technologies) using the same fluorescence parameters as described for the HPLC method above.

### 2.7. Detection of Mitochondrial ROS Formation in Isolated Heart Mitochondria

For detection of mitochondrial ROS formation, a published protocol was used [36,41]. Mitochondria were isolated from the hearts from sham-treated rats as previously described above. We diluted the suspensions of mitochondria in 0.5 mL of PBS buffer containing L-012 (100 µM) (final protein concentration: 0.1 mg/mL). We stimulated the generation of ROS with succinate (final concentration: 5 mM) and with myxothiazol or antimycin A (final concentrations: 10 µM or 10 µg/mL). In some cases, the L-012 ECL signal was inhibited by ROS scavenging using the manganese-porphyrin (MnTMPyP, 10 µM). The chemiluminescence was registered at intervals of 30 s over 5 min using a Mithras^2^ chemiluminescence plate reader (Berthold Technologies, Bad Wildbad, Germany) and the signal was expressed as counts/min at 5 min.

### 2.8. Statistical Analysis

Results are expressed as means ± SD. We used Two-way ANOVA (with Bonferroni’s correction for comparison of multiple means) for comparative analysis of isometric tension recording data, expressed by the concentration-relaxation curves (Prism for Windows, version 7.01, GraphPad Software Inc., San Diego, CA, USA). We applied One-way ANOVA (with Bonferroni’s correction for comparison of multiple means) or, where appropriate, respective non-parametric Kruskal-Wallis test (with Dunn’s correction for comparison of multiple means) for comparative analysis of ROS detection, oxidative protein modification and protein expression (SigmaStat for Windows, version 3.5, Systat Software Inc., San Jose, CA, USA). *p* values < 0.05 were considered statistically significant. As a limitation of the study we would like to mention that the sham-treated control rats were summarized in one control group since only marginal (non-significant) differences were observed (NaCl or ethanol infusion for comparison with hypertensive or nitrate tolerant rats as well as citrate buffer injection for comparison with diabetic rats).

## 3. Results

### 3.1. Detection of Superoxide Generation by Xanthine Oxidase Using mitoSOX HPLC

**Figure 2:**
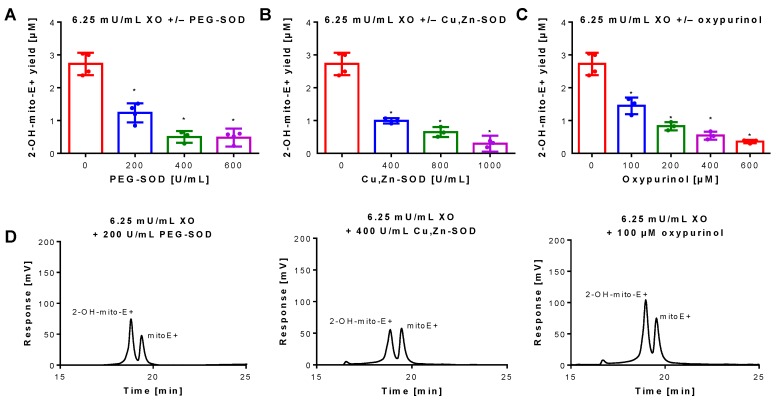
Inhibition of superoxide formation and detection by mitoSOX HPLC. An XO concentration of 6.25 mU/mL was chosen for continuous and sufficient superoxide formation. The yield of the superoxide-specific mitoSOX oxidation product 2-OH-mito-E+ was quantified in the presence of increasing concentrations of the superoxide scavenging enzymes PEG-SOD or native Cu, Zn-SOD (**A**,**B**) or the XO inhibitor oxypurinol (**C**). The reaction solution contained 0.1 M potassium phosphate buffer at pH 7.4 and 1 mM hypoxanthine and was incubated for 30 min at 37 °C. (**D**) Representative chromatograms are shown for the lowest inhibitor concentration. Data are mean ± SD of 3–4 independent experiments. * *p* < 0.05 vs. unstimulated control.

Superoxide generation by xanthine oxidase (XO) was quantified by 2-OH-mito-E+ concentrations using HPLC analysis. The 2-OH-mito-E+ yield showed a plateau at 25 mU/mL XO indicating that all mitoSOX dye (5 µM) was oxidized to the 2-OH-mito-E+ and other products (Figure 1B). The 2-OH-mito-E+ yield showed an almost linear correlation with XO concentrations up to 6.25 mU/mL (Figure 1C), with a calculated superoxide formation rate of 1.15 µM/min by 6.25 mU/mL XO [29]. The specificity of the assay for superoxide detection was shown by concentration-dependent scavenging of the 2-OH-mito-E+ yield by PEG-SOD or Cu, Zn-SOD (Figure 2A,B). The XO inhibitor oxypurinol also decreased the 2-OH-mito-E+ yield at higher concentrations (Figure 2C). We would like to mention that during the incubation time of 30 min at 37 °C (and probably the storage at 4 °C in the autosampler before measurement) we observed substantial autoxidation of mitoSOX without added XO yielding approximately 1 µM of 2-OH-mito-E+. However, it should be noted that XO even at low concentrations clearly caused additive 2-OH-mito-E+ generation on top of this “background” and having mitoSOX in pure buffer is rather artificial since oxygen concentrations in biological samples are usually lower and biological fluids contain antioxidants that prevent autoxidation (as seen in our subsequent experiments).

### 3.2. Detection of Superoxide Generation by Isolated Rat Heart Mitochondria Using mitoSOX HPLC

**Figure 3:**
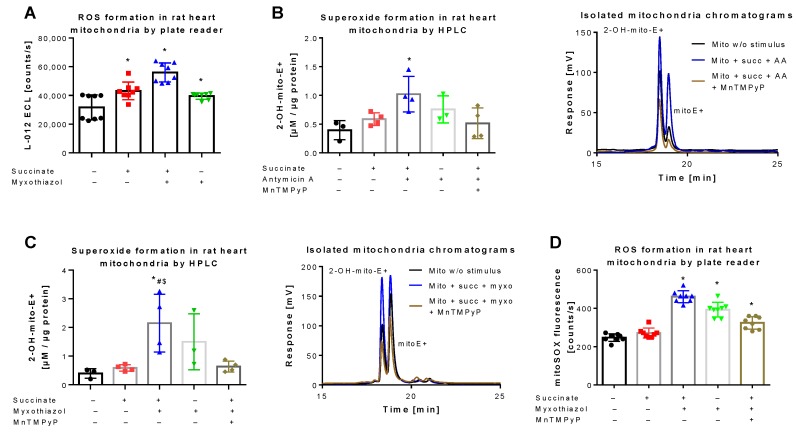
Detection of superoxide generation by mitochondria by mitoSOX HPLC. (**A**) Quantification of reactive oxygen species (ROS) formation by L-012 (100 µM) ECL in response to different stimuli. The reaction solutions contained PBS buffer at pH 7.4, 5 mM succinate, 10 µM myxothiazol or 10 µg/mL antomycin A and was incubated for 5 min at 37 °C. Using similar conditions the superoxide formation was detected by HPLC-based quantification of 2-OH-mito-E+ (**B**,**C**) and ROS formation was measured using a fluorescence plate reader assay for the mitoSOX oxidation products (**D**), both also in the presence of 20 µM MnTMPyP as a superoxide scavenger. Representative chromatograms are shown for the HPLC-based quantification of 2-OH-mito-E+ (**B**,**C**). Data are mean ± SD of 8 (**A**), 3–4 (**B**,**C**) and 8 (**D**) independent experiments. * *p* < 0.05 vs. unstimulated control; ^#^ *p* < 0.05 vs. succinate alone group; ^$^ *p* < 0.05 vs. MnTMPyP group.

L-012 oxidation represents a classical chemiluminescence-based ROS detection assay that can be used for qualitative quantification of oxidative stress in mitochondria [36,42]. L-012 chemiluminescence showed a significant increase in signal upon stimulation of mitochondrial respiration at complex II by succinate, which was further augmented in the presence of the complex III inhibitor myxothiazol (Figure 3A). A quite similar signal pattern was observed when superoxide formation in isolated mitochondria was quantified by HPLC-based 2-OH-mito-E+ detection. Succinate and myxothiazol or antimycin A increased the 2-OH-mito-E+ yield, whereas addition of the manganese superoxide dismutase mimetic MnTMPyP decreased it to the level of control (Figure 3B,C). Under these conditions, the 2-OH-mito-E+ yield could also be quantified using a plate reader assay, without discrimination between the different mitoSOX oxidation products, again generating a similar signal pattern (Figure 3D). However, the absolute differences observed with the plate reader assays (L-012 and mitoSOX) were somewhat less pronounced than with the HPLC-based method, probably due to high background coming from unspecific oxidation of the dye, clearly identifying the mitoSOX/HPLC method as the most sensitive one for detection of small differences in mitochondrial superoxide formation. Different protein concentrations of the used mitochondrial preparations did not show a linear correlation with the 2-OH-mito-E+ yield since a 10-fold increase in protein concentration only caused a 3.5-fold increase in 2-OH-mito-E+ yield (not shown), which could be related to incomplete extraction of the oxidized products at higher protein content, more pronounced scavenging by antioxidant moieties of mitochondria (e.g., Fe-sulfur-clusters or reactive thiols) or too high superoxide formation rates with reaching the plateau of oxidized products.

### 3.3. Detection of Superoxide Generation by Isolated Rat Heart Mitochondria From Rats with Preestablished Oxidative Stress Using mitoSOX HPLC

**Figure 4:**
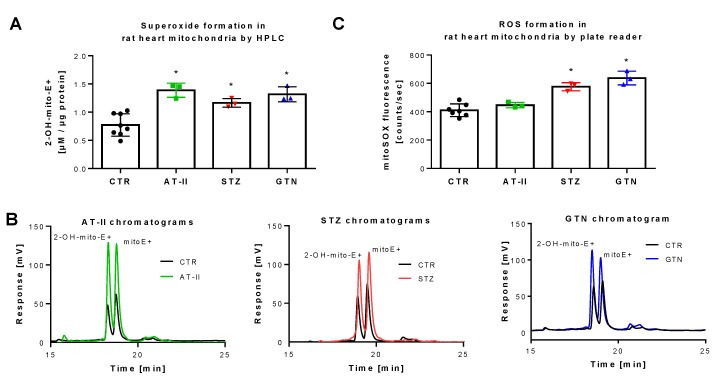
Detection of superoxide generation by mitochondria from rats with preestablished cardiovascular disease or pharmacologically induced oxidative stress by mitoSOX HPLC. The yield of the superoxide-specific mitoSOX oxidation product 2-OH-mito-E+ in mitochondrial preparations of hypertensive (AT-II), diabetic (STZ) and nitrate tolerant rats (GTN), and respective control animals (**A**). Representative chromatograms are shown for the HPLC-based quantification of 2-OH-mito-E+ (**B**). In the same mitochondrial preparations, also ROS formation was measured using a fluorescence plate reader assay for the mitoSOX oxidation products in all groups (**C**). Data are mean ± SD of 7 (Ctr) and 3 (AT-II, STZ, GTN) rats per group. Each single animal data point was generated from up to 2 (**A**) or 2–3 (**C**) technical replicates. * *p* < 0.05 vs. control group.

The rat models used for these measurements were previously reported to display severe oxidative stress. Induction of hypertension by AT-II infusion [43,44,45], of diabetes by STZ injection [46,47,48] and of nitrate tolerance by GTN treatment [28,35,49] all share the activation of vascular NADPH oxidases and mitochondrial superoxide formation. Applying HPLC analysis, we found increased 2-OH-mito-E+ yields in isolated mitochondria of these three animal models as compared to control animals (Figure 4A,B). When the plate reader method was used for quantification of mitoSOX oxidation products, a similar signal pattern was observed but the absolute differences were much lower than those found by the HPLC method (Figure 4C), which again may be explained by the high background coming from unspecific oxidation of the dye, clearly identifying the mitoSOX/HPLC method as the most sensitive one for detection of small differences in mitochondrial superoxide formation. Of note, the different animal disease models were investigated on different time points (several weeks in between) and we, therefore, cannot conclude that mitoSOX/HPLC data allow the comparison of the absolute mitochondrial superoxide levels values between the different disease models (this would have required ideally an intraday comparison of these groups) but reflects that all three disease models are associated with increased mitochondrial superoxide formation.

### 3.4. Comparison of mitoSOX HPLC Results with Vascular Function and Other Oxidative Stress Markers

**Figure 5:**
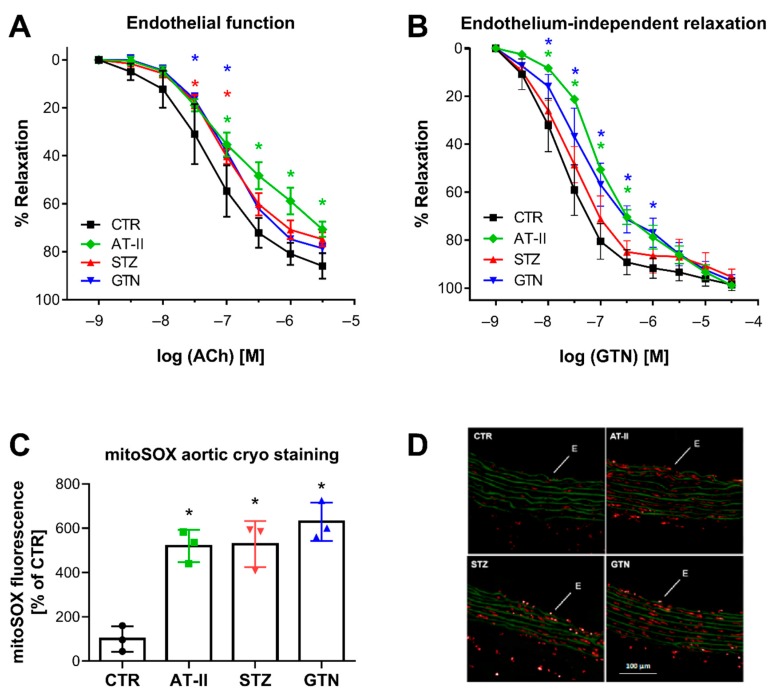
Vascular function and ROS formation of aorta from rats with preestablished cardiovascular disease or pharmacologically induced oxidative stress. Endothelium-dependent relaxation (ACh-triggered) (**A**) and endothelium-independent relaxation (GTN-triggered) (**B**) was determined by isometric tension method in aortic ring segments of hypertensive, diabetic and nitrate tolerant rats, and respective control animals. (**C**) Vascular ROS formation was determined by mitoSOX oxidative fluorescent microtopography in aortic cryo-sections. Representative fluorescence images are shown with red fluorescence for mitoSOX oxidation products and green fluorescence for autofluorescence of the basal laminae (**D**). Data are mean ± SD of 7 (Ctr, **A**,**B**), 3 (AT-II, STZ, GTN, A,B) and 3 (**C**) rats per group. Each single animal data point was generated from 3–4 (**A**,**B**) or 3 (**C**) technical replicates. * *p* < 0.05 vs. control group.

There was a nice correlation between HPLC-based 2-OH-mito-E+ yields and vascular function parameters. Endothelial function (acetylcholine-dependent relaxation) was measured in aorta and was significantly impaired in the hypertensive, diabetic and nitrate tolerant rats (Figure 5A). The endothelium-independent relaxation (GTN-triggered) was impaired in hypertensive and nitrate tolerant rats, indicating a significant degree of nitrate tolerance (in the case of GTN-treated rats) or nitrate resistance (in the case of hypertensive rats) (Figure 5B). Oxidative fluorescent microtopography represents a classical fluorescence-based ROS detection assay that can be used for spatial distribution of ROS formation in tissue cryo-sections, thereby providing information of the cellular localization of the ROS sources. We found that mitoSOX oxidative fluorescent microtopography can be used in aortic cryo-sections for detection of ROS formation since the fluorescence signal by oxidized mitoSOX products was higher in the aorta of the three disease models then in those of control rats (Figure 5C,D). Therefore, mitoSOX oxidative fluorescent microtopography may be a valuable method for detection of mitochondrial ROS formation when tissue material is limited as in the case of aorta and hence isolation of mitochondria is technically sophisticated. It may be combined with PEG-SOD or mitoTEMPO (2,2,6,6-tetramethyl-4-[[2-(triphenylphosphonio)acetyl]amino]-1-piperidinyloxy) to test for mitochondrial superoxide formation.

**Figure 6:**
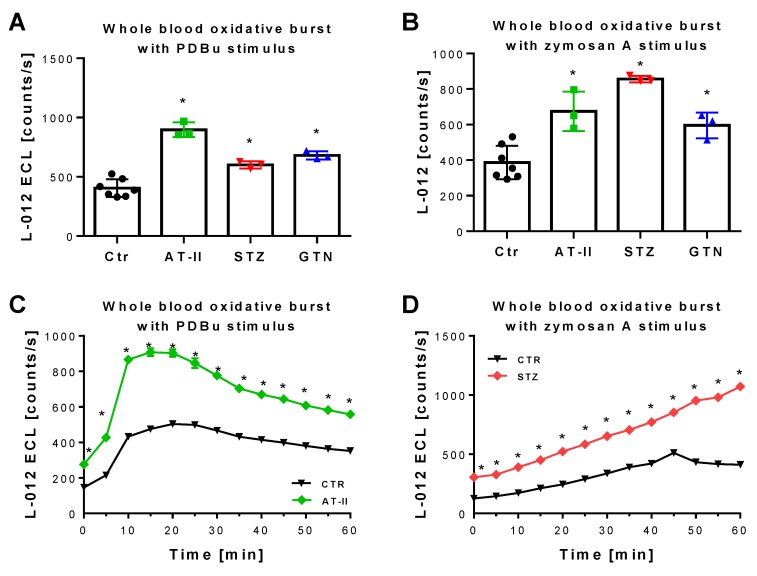
Detection of ROS formation during oxidative burst in whole blood from rats with preestablished cardiovascular disease or pharmacologically induced oxidative stress. Quantification of ROS formation by L-012 (100 µM) ECL in response to different stimuli. The reaction solutions contained blood 1:50 in PBS buffer at pH 7.4, 10 µM PDBu (**A**) or 10 µg/mL zymosan A (**B**) and was incubated for 15 min (PDBu) or 45 min (zymosan A) at 37 °C. Representative kinetic traces are shown for 1 animal per group upon PDBu (Ctr vs. AT-II) or zymosan A (Ctr vs. STZ) stimulation with 4 technical replicates per data point (**C**,**D**). Data are mean ± SD of 7 (Ctr, and 3 (AT-II, STZ, GTN) rats per group. Each single animal data point was generated from 2–4 technical replicates (**A**,**B**). * *p* < 0.05 vs. control group.

**Figure 7:**
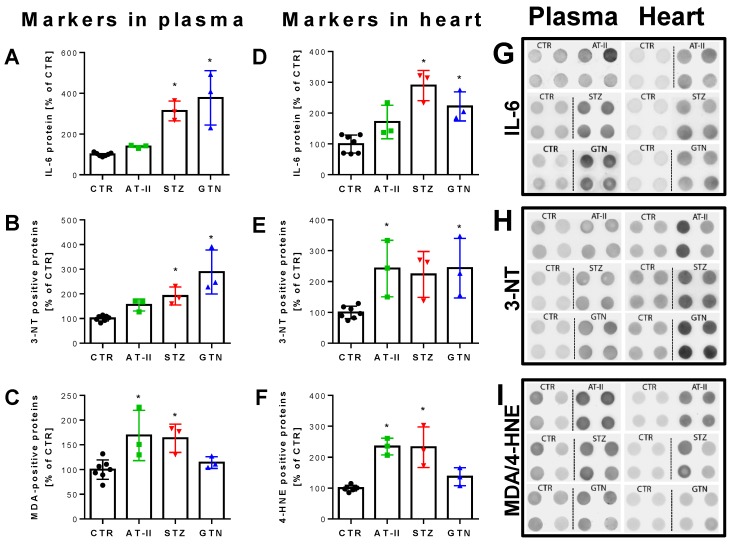
Biomarkers in plasma and heart from rats with preestablished cardiovascular disease or pharmacologically induced oxidative stress. Markers of oxidative stress (3-NT, MDA, 4-HNE) and inflammation (IL-6) were increased in plasma (**A**–**C**) and heart (**D**–**F**) of hypertensive, diabetic and nitrate tolerant rats as compared to control animals. Original blots are shown on the right for 4 technical replicates per group (**G**–**I**). Data are mean ± SD of 7 (Ctr) and 3 (AT-II, STZ, GTN) rats per group. Each single animal data point was generated from 2–4 technical replicates. * *p* < 0.05 vs. control group.

The 2-OH-mito-E+ yields measured by HPLC also correlated with the oxidative burst signals detected by L-012 chemiluminescence in whole blood. This classical ROS detection assay reflects NADPH oxidase activity in leukocytes (NOX-2 isoform) during oxidative burst upon maximal stimulation by phorbol esters or endotoxins [37] and requires the presence of hydrogen peroxide and peroxidases [50], both present in leukocytes. Whole blood oxidative burst was increased in hypertensive, diabetic and nitrate tolerant rats, regardless of the stimulus (Figure 6A,B). Finally, classical well established oxidative stress markers such as proteins that are positive in immunostaining for 3-nitrotyrosine (3-NT, footprint of peroxynitrite), malondialdehyde and 4-hydroxynonenal (MDA and 4-HNE, footprints of lipid peroxidation) were determined in plasma and heart tissue using dot blot analysis. The majority of these markers was clearly positive in the disease animal models or at least showed a trend of increase (Figure 7A–F). Since oxidative stress is a hallmark of inflammation, which represents an important pathomechanism for the development and progression of cardiovascular disease, we also measured the inflammation marker IL-6 that was increased, at least by trend, in all of the used disease animal models.

## 4. Discussion

With the present study, we show that mitoSOX/HPLC reflects mitochondrial superoxide formation in diabetic, hypertensive and nitrate tolerant rats. Increased mitoSOX oxidation inversely correlated with vascular function and was supported by established markers of oxidative stress and classical RONS detection assays. The superoxide-specific mitoSOX oxidation product 2-OH-mito-E+ showed a linear increase over an appreciable concentration range of xanthine oxidase and the signal was highly sensitive to superoxide scavengers such as PEG-SOD or MnTMPyP. In isolated rat heart mitochondria, the superoxide-specific mitoSOX oxidation product 2-OH-mito-E+ was increased by complex III inhibitors myxothiazol or antimycin A in the presence of succinate, which was prevented by MnTMPyP. Qualitatively similar results were obtained with the L-012 enhanced chemiluminescence assay.

Why is the quantification of mitochondrial superoxide formation of special interest? First, there are numerous redox-sensitive structures in the mitochondrial matrix and intermembrane space such as iron-sulfur clusters, thiol-rich enzymes and redox regulated mitochondrial pores [21,51]. Accordingly, mitochondrial RONS formation can lead to substantial functional damage. Second, as already mentioned in the introduction, the knowledge on the spatial distribution of RONS formation may be of specific interest for drug development and when it comes to therapy [52]. An example is the direct and specific targeting of mitochondrial RONS formation by mitochondria-targeted antioxidants, which will decrease mitochondrial RONS formation and prevent oxidative damage of important mitochondrial structures (e.g., mitochondrial DNA is not well protected since efficient DNA repair systems do not exist in the matrix) [53]. Examples for mitochondria-targeted antioxidants that are widely used are mitoQ ([10-(2,5-dihydroxy-3,4-dimethoxy-6-methylphenyl)decyl]triphenyl-phosphonium) [54], mitoTEMPO (2,2,6,6-tetramethyl-4-[[2-(triphenylphosphonio)acetyl]amino]-1-piperidinyloxy) [55] or the peptide SS-31 (d-Arg-2′,6′-dimethyltyrosine-Lys-Phe-NH_2_) [56]. Third, according to the “redox-crosstalk concept” between all enzymatic sources of RONS [57,58], mitochondria represent a central hub within this network and either provide the kindling radicals that activate other sources of RONS such as NADPH oxidases, xanthine oxidase or uncoupled nitric oxide synthase or act as amplifiers of RONS produced by cytosolic enzymes via numerous mechanisms [21,59]. A central role of mitochondrial RONS formation and vital crosstalk between different sources of RONS was demonstrated for the pathogenesis of hypertension [45,55] and nitrate tolerance [29]. This redox-crosstalk can also be extended to inflammation [20] since mitochondrial RONS can directly stimulate Toll-like receptor signaling and bactericidal activity of macrophages [60] and cause activation of the Nod-like receptor protein family pyrin domain containing 3 (NLRP3) inflammasome [61].

Most previous studies using mitoSOX either stained cells with the dye for subsequent flow cytometry [62,63,64], stained tissue sections in combination with fluorescence microscopy [65,66] or simply stained cultured cells or mitochondrial suspensions in combination with a fluorescence plate reader [67]. However, thereby the mitoSOX signal will probably lose the superoxide-specificity since these methods cannot distinguish between the various oxidation products of mitoSOX, a feature that is only provided by mitoSOX/HPLC [68,69], although optimized wavelength setting have been reported for fluorescence microscopy to increase the specifity for superoxide oxidation product [70,71]. A few reports on mitoSOX/HPLC used isolated mitochondria [31] or cells [72,73] for detection of mitochondrial superoxide formation. Even less studies applied mitoSOX/HPLC to tissue samples to reveal the mitochondrial superoxide formation differences. This was reported for aortic tissue of wildtype versus AMPK knockout mice [74] or mitochondria from cardiac tissue of rats with myocardial infarction [75]. The rather limited use of mitoSOX/HPLC method may be also related to reports indicating that hydroethidium-based probes may undergo time-dependent autoxidation in the presence of oxygen (and obviously accelerated by light exposure) [30], although when coming from the vendor, the dye is quite stable (see Materials and Methods Section) and we do not know much about the stability of the mitoE+ and 2-OH-mito-E+ oxidation products against autoxidation. In addition, the fluorescence intensity of all DHE derivatives in biological samples changes with intercalation with DNA [69,71]. Accordingly, the amount of DNA in a given samples will not only influence the signal intensity but may also change the stability of the educts and products, although not much is known on the impact of DNA intercalation on the stability of DHE compounds, e.g., towards autoxidation. Also the fact that higher mitoSOX concentrations themselves can cause disruption of mitochondrial membrane potential and uncoupling of mitochondrial respiration with higher ROS formation [64] may prevent a broader use of mitoSOX. Kauffman and colleagues not only showed that mitochondrial superoxide formation in general and mitoSOX signal in particular rely on the presence of mtDNA (encoding for respiration complexes) but also showed a similar pattern of ROS formation comparing mitoSOX and lucigenin [64].

In light of these previous results our present study is unique, not only because we applied mitoSOX/HPLC to three animal disease models of oxidative stress and found higher signals of 2-OH-mito-E+ in all diseased tissues, but also since we can link the increased mitochondrial superoxide levels with adverse functional effects such as endothelial dysfunction or nitrate tolerance/resistance and we can demonstrate that mitoSOX oxidation goes hand in hand with an increase in other markers of oxidative stress such as 3-nitrotyrosine, malondialdehyde or 4-hydroxynonenal in plasma and/or heart samples. In addition, we provide evidence that increased mitochondrial oxidative stress is associated with higher levels of the inflammatory marker IL-6, thereby supporting the redox-crosstalk of mitochondrial RONS and inflammation. Finally, we support the postulated loss of specificity of the mitoSOX signal, when using plate reader assay [68], as we detected much lower absolute differences using the mitoSOX fluorescence plate reader assay as compared to mitoSOX/HPLC. 

## 5. Conclusions

In conclusion, we report that mitoSOX/HPLC indeed represents a suitable method for quantification of mitochondrial superoxide formation in biological samples of animals with cardiometabolic disease or pharmacologically induced cardiovascular complications. Moreover, mitoSOX/HPLC correlates with functional and other biochemical and molecular biological oxidative stress/inflammation parameters and has predictive value for adverse cardiovascular phenotypes. Of note, also the classical (old) assays for RONS detection such as L-012 ECL plate reader assay in isolated mitochondria or whole blood as well as mitoSOX fluorescence plate reader assay in isolated mitochondria or microscopy in aortic cryo-sections showed similar correlations and predictive value in this study but mitoSOX HPLC is superior with respect to absolute differences between control and diseased animals as well as specificity for mitochondrial superoxide. Thereby, our present data also nicely add to the ongoing discussion whether hydroethidium-based probes may be used for superoxide detection in cell culture [30] or even tissue samples. Finally, we would like to emphasize that the autoxidation of mitoSOX is an inevitable side effect when working at ambient oxygen concentrations but that matching the incubation times as well as storage until measurement will cause a comparable background in all samples and additive 2-OH-mito-E+ generation on top of this “background” will allow specific detection of source-dependent superoxide formation. In addition, incubation of mitoSOX in pure buffer is rather artificial since oxygen concentrations in biological samples are usually lower and biological fluids contain antioxidants that prevent autoxidation. We would also like to stress that mitoSOX/HPLC data does not allow the comparison of the absolute mitochondrial superoxide levels values between the different disease models when animals were not investigated on the same day (intraday comparison of groups is required) but allows the conclusion on increased mitochondrial superoxide formation in all three animal disease models.
